# Online-rotation in paediatrics – digital live-interaction with children

**DOI:** 10.3205/zma001394

**Published:** 2020-12-03

**Authors:** Martina Peter-Kern, Christoph Härtel, Sarah König

**Affiliations:** 1 Universitätsklinikum Würzburg, Kinderklinik und Kinderpoliklinik, Würzburg, Germany; 2 Universität Würzburg, Institut für Medizinische Lehre und Ausbildungsforschung, Würzburg, Germany

**Keywords:** online block rotation, digitalization, teaching during the SARS-CoV-2 pandemic, online teaching

## Abstract

The two-week block rotation in paediatrics (tenth semester) took place for 62 students purely as online teaching in the summer semester of 2020, at the time of the initial restrictions. As a teaching module, virtual patient presentations including debriefing took place as synchronous teaching. Patients and one parent were broadcast from the wards and outpatient clinics via video conference. Students were able to interact in small groups with 15-22 patients or their parents, respectively, via a doctor and both conduct the case history interview and brief the examination steps. Despite the limitation of not being able to perform the clinical examination themselves, participants rated the block rotation with good marks. They particularly appreciated the ability to interact with the children online as an indispensable compromise in times of suspended classroom teaching during the SARS-CoV-2 pandemic.

## Zusammenfassung

Das zweiwöchige Blockpraktikum Pädiatrie (10. Semester) fand für 62 Studierende im Sommersemester 2020 zur Zeit der Ausgangsbeschränkung als reiner Online-Unterricht statt. Als ein Unterrichtsbaustein fanden virtuelle Patientenvorstellungen inkl. Nachbesprechung als synchrone Lehre statt. Patientinnen und Patienten sowie ein Elternteil wurden von den Stationen und Ambulanzen per Videokonferenz übertragen. Die Studierenden konnten in Kleingruppen über eine ärztliche Person mit jeweils 15-22 Patienten bzw. deren Eltern interagieren und sowohl das Anamnesegespräch führen, also auch Untersuchungsschritte instruieren. Die Teilnehmenden bewerteten das Blockpraktikum trotz der Limitierung, nicht selbst die klinische Untersuchung durchführen zu können, mit guten Noten und wertschätzten insbesondere die Möglichkeit zur Online-Interaktion mit den Kindern als unverzichtbaren Kompromiss in Zeiten der ausgesetzten Präsenzlehre während der SARS-CoV-2-Pandemie.

## Background

The SARS-CoV-2 pandemic posed significant challenges for teachers of clinical medicine [1]. All of a sudden, proven face-to-face teaching had to be transformed into an online format. While this seemed feasible for digitally converted lectures and seminars and appeared to serve a certain “zeitgeist” particularly with regard to knowledge transfer, elaborate digital alternatives had to be developed for training involving direct contact with patients. Accordingly, the paediatrics block rotation, which usually requires two weeks of attendance on ward, was converted into a purely online format, with virtual patient rounds as a key feature. The goal was to enable the applicability of the subject through personal patient contact, thus training clinical observational skills and communication with children and to investigate the question as to how far this may be achieved to the satisfaction of the participants.

## Method

**Figure 1:**
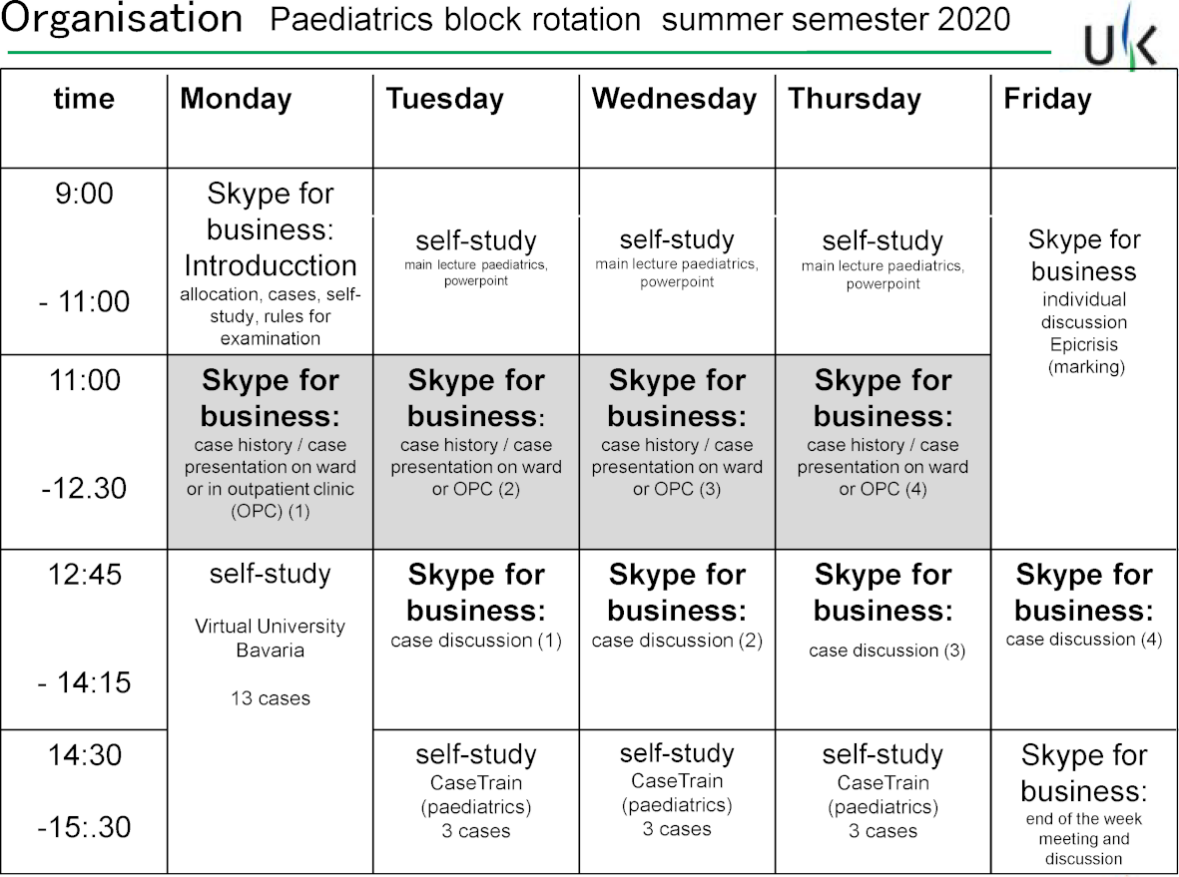
Timetable for the online rotation paediatrics as a one-week example

The two-week block rotation in paediatrics (tenth semester) took place for 62 students purely as online teaching in the summer semester of 2020, at the time of the initial restrictions. In this best practice example, we utilized the video conferencing platform “Skype for Business” (Microsoft, USA). One of the children's parents agreed in writing to the broadcast for the purposes of medical education. The patients were broadcast from wards and outpatient clinics via mobile devices using both cameras and microphones. A medical teacher and another technical assistant were present on site in the clinic, while students joined in groups of 12-16 participants for scheduled sessions (see figure 1 (Fig. 1), timetable exemplary of one week). The students performed a full case history interview not only focussing on current complaint and history of the presenting illness. In addition, participants were able to gain insight into their patients’ general condition, understand externally visible findings, and thus practice visual diagnosis. During the physical examination, the cooperation of the students was also required in that they described the necessary examination steps to the teacher in-situ, analogous to the third step of the Peyton method [2], before the teacher implemented them. Outside the patient's room, a round of discussions on further diagnostics, therapy, prognosis, and epidemiology followed. 

Each virtual patient presentation, including debriefing, lasted 90 minutes. They took place four times a week for each student, involving up to three children per session. During the remaining time of the block rotation, there were further digital training elements. For example, students received by e-mail findings extracted from patient files, which they used to work out case presentations to be held in web meetings. There were also lecture recordings and e-learning cases (CaseTrain [3]). As examination of their performance, students were tasked with writing an epicrisis of an example case (with a given medical history, laboratory findings, and examination results).

In a final online evaluation (EvaSys®, Lüneburg, Germany), students could assess their satisfaction and personal learning success, as well as comment on the online alternative of the block rotation in the form of free-text answers.

## Results

The digital block rotation enabled each participant to interact with 15-22 children and their parents. Whereas the preferred group of 3-16 year-olds selected for the patient presentation had little inhibition towards interacting via a monitor, this was hardly possible with younger children and was therefore replaced by conversations with the parents. In addition, pre-recorded video footage taught infant examination procedures with discussion of the steps in online lessons. Students were able to conduct interviews in a paediatric context and brief the examination steps. In addition, they were actively challenged to work on patient cases and thus transfer their theoretical knowledge to the clinical context and deal with clinical decision-making. 

Forty-two students participated in the evaluation (response rate 68%). A large majority (89.5 %) of the participants affirmed the item “I was satisfied with the virtual patient presentation module”. The item “I learned a lot” was rated with an average value of 1.95 (on the scale 1=very good to 5=poor). In the free-text comments, it became clear that students found the live interaction with the young patients and their parents particularly valuable, and viewed the experience as a good compromise within the framework of online teaching. The vast majority of students expressed that they would recommend the online module to others. Students nevertheless regretted the inevitable fact that they were unable to examine the children themselves. 

Teaching staff were aware of this limitation as well. When the module was carried out, it also became apparent that the personnel required during the online paediatric block rotation significantly exceeded that of the usual format with “students on site”. 

## Conclusion

In view of the suspension of face-to-face teaching, the online block rotation in paediatrics with the virtual ward and outpatient clinic rounds proved to be an alternative option to real-world patient contact. 

Interaction via video mainly served communication and the viewing of clinical findings of sick children and was naturally dependent on a fundamental willingness to communicate as well as age-dependent ability to cooperate. The online format could not replace the students’ own haptic experience of performing an examination themselves. Nevertheless, under the given conditions and with good case and patient selection, this concept can be instructive and effective as a substitute in the sense of case-based training [4], [5]. To what extent online patient presentation, supplemented by further digital teaching, can or must prove itself in the long run will be demonstrated by the teaching conditions resulting from the development of the epidemiological situation of SARS-CoV-2 infections.

## Competing interests

The authors declare that they have no competing interests. 

## References

[1] Ashokka B, Ong SY, Tay KH, Loh NH, Gee CF, Samarasekera DD (2020:42(7)). Coordinated responses of academic medical centres to pandemics: Sustaining medical education during COVID-19. Med Teach.

[2] Krautter M, Dittrich R, Safi A, Krautter J, Maatouk I, Moeltner A, Herzog W, Nikendei C (2015). Peyton's four-step approach: differential effects of single instructional steps on procedural and memory performance - a clarification study. Adv Med Educ Pract.

[3] Hörnlein A, Ifland M, Kluegl P, Puppe F (2009). Konzeption und Evaluation eines fallbasierten Trainingssystems im universitätsweiten Einsatz (CaseTrain). GMS Med Inform Biom Epidemiol.

[4] Reed S, Shell R, Kassis K, Tartaglia K, Wallihan R, Smith K, Hurtubise L, Martin B, Ledford C, Bradbury S, Bernstein HH, Mahan JD (2014). Applying adult learning practices in medical education. Curr Probl Pediatr Adolesc Health Care.

[5] Ali M, Han SC, Bilal HS, Lee S, Kang MJ, Kang BH, Razzaq MA, Amin MB (2018). iCBLS: An interactive case-based learning system for medical education. Int J Med Inform.

